# Solar Cycle Variation of the Distribution of Photospheric Magnetic Flux Features

**DOI:** 10.1007/s11207-026-02715-0

**Published:** 2026-08-21

**Authors:** Callan N. Noble, Clare E. Parnell, Thomas Neukirch

**Affiliations:** School of Mathematics and Statistics, University of St Andrews, North Haugh, St Andrews, KY16 9SS UK

**Keywords:** Magnetic fields, Photosphere, Solar cycle, Observations, Sunspots, Statistics

## Abstract

We use statistical tools to analyse data from the Solar Dynamics Observatory Helioseismic and Magnetic Imager to determine the distribution of the magnetic flux of photospheric magnetic features and its variation over a full solar cycle. In particular, we use statistical figures of merit to test how well different types of probability density function represent the magnetic flux distribution inferred from the data and how their shape changes over the solar cycle. Our analysis shows that a double power law provides the best representation of the data over the full solar cycle and we present the dependence of the power law exponents on the phase of the solar cycle. The nature of the observed flux distributions at different times during the solar cycle is significant because it could be used to try and infer information about solar magnetic field generation mechanisms. We discuss potential implications of a double power law distribution for solar magnetic field generation.

## Introduction

Improving our understanding of the spatiotemporal behaviour of the distribution of magnetic flux on the solar surface is important for several areas of solar physics. On the one hand, the characteristics of the solar surface magnetic field can provide insights into the dynamo processes taking place in the Sun’s interior (e.g. Brandenburg, Sokoloff, and Subramanian 2012; Charbonneau 2014, 2020; Brun and Browning 2017; Rempel et al. 2023). On the other hand, with accurate measurements of the solar magnetic field currently only possible at photospheric levels (e.g. Bellot Rubio and Orozco Suárez 2019; Wiegelmann and Sakurai 2021), the structure and dynamics of the photospheric magnetic flux also provides a crucial input into our understanding of the structure of the coronal magnetic field and phenomena like coronal heating and magnetic activity processes (e.g. Mackay and Yeates 2012; Petrie 2013; Pevtsov et al. 2021).

Because it involves the largest scales, the most obvious manifestation of the processes generating the solar magnetic field in the solar interior is the 11-year cycle in sunspot numbers, which is a 22-year cycle if the polarity of magnetic fields is taken into account (e.g. Solanki, Inhester, and Schüssler 2006; Hathaway 2015). While sunspots are observed in the active-region bands between $\pm 40^{\circ}$ latitude, magnetic features of all sizes can be observed on the solar surface and smaller magnetic features seem to be distributed across the entire solar surface (e.g. Solanki, Inhester, and Schüssler 2006).

Due to their general importance, different aspects of the solar surface magnetic field and its characteristics have been investigated in the past. Numerous studies focus on larger magnetic features such as pores, sunspot umbrae, sunspots and sunspot groups. Observations have shown a strong correlation between the magnetic flux and area of sunspots, as well as active regions in general, (e.g. Nicholson 1933; Houtgast and van Sluiters 1948; Pevtsov et al. 2014; Nagovitsyn, Pevtsov, and Osipova 2017; Pevtsov et al. 2021; Wang, Jiang, and Luo 2023) and hence the distribution of both of these quantities, as well as active regions in general, has been investigated intensively (e.g. Kuklin 1980; Bogdan et al. 1988; Baumann and Solanki 2005; Zharkov, Zharkova, and Ipson 2005; Schad and Penn 2010; Jiang et al. 2011; Nagovitsyn, Pevtsov, and Livingston 2012; Cho et al. 2015; Muñoz-Jaramillo et al. 2015; Kostyuchenko 2017; Nikbakhsh et al. 2019; Tlatov, Riehokainen, and Tlatova 2019; Nagovitsyn and Pevtsov 2021; Sakurai and Toriumi 2023; Kumar, Kumar, and Vats 2025). Other studies have focussed on bipolar magnetic regions (e.g. Tang, Howard, and Adkins 1984; Harvey and Zwaan 1993; Zhang, Wang, and Liu 2010), the quiet network magnetic flux (e.g. Schrijver et al. 1997), ephemeral regions (e.g. Parnell 2002), or emerging flux features (e.g. Thornton and Parnell 2011).

Instead of considering either larger scale features or a specific set of features there have also been a number of studies of the distribution of features across all scales. For example, Meunier (2003) used SOHO/MDI full-disk magnetograms to examine the statistical distribution of the magnetic flux and the variation of this distribution, which they found to be a power law, with the solar cycle. Parnell et al. (2009) combined SOHO/MDI data with observations by Hinode/SOT, which allowed them to cover five orders of magnitude in flux. These authors also found that the magnetic flux distribution is represented by a single power law. A more recent study of a similar type, but concentrating on quiet sun flux elements, has been undertaken by Javaherian et al. (2017) on the basis of SDO/HMI magnetograms, with the authors finding a broken (or double) power law distribution function. Depending on the data sets and features studied, very different forms for the distribution functions have been put forward, for example exponential distributions (e.g. Tang, Howard, and Adkins 1984; Schrijver et al. 1997), log-normal distributions (e.g. Bogdan et al. 1988; Baumann and Solanki 2005; Zhang, Wang, and Liu 2010; Schad and Penn 2010), double log-normal distributions (Kuklin 1980; Nagovitsyn, Pevtsov, and Livingston 2012; Nagovitsyn and Pevtsov 2021, e.g.), or, as already mentioned above, power law distributions (e.g. Zharkov, Zharkova, and Ipson 2005; Meunier 2003; Parnell et al. 2009; Thornton and Parnell 2011; Shapoval et al. 2018). Other forms of suggested distribution functions are polynomials (e.g. Harvey and Zwaan 1993), the Weibull distribution (e.g. Parnell 2002), broken (or double) power law distributions (e.g. Javaherian et al. 2017; Song et al. 2024), and bi-modal log-normal functions (e.g. Cho et al. 2015), as well as combinations of distribution functions such as power law and log-normal (e.g. Jiang et al. 2011) and Weibull and log-normal (e.g. Muñoz-Jaramillo et al. 2015). The distribution functions mentioned above can be split into to different classes: unimodal distributions such as, for example a power-law distribution, and bimodal distributions like the hybrid distributions, broken/double power laws or double log-normal distributions. Bimodal distributions are often regarded as being indicative of two different physical processes generating the distinct parts of the distribution (e.g. Nagovitsyn, Pevtsov, and Livingston 2012; Muñoz-Jaramillo et al. 2015; Song et al. 2024) or that it could be representing different stages in the evolution of certain magnetic features (e.g. Tlatov, Riehokainen, and Tlatova 2019). On the other hand, unimodal distributions (e.g. Schrijver et al. 1997; Parnell et al. 2009) could be indicative of a single physical process operating over all scales of the distribution.

In this paper we carry out a statistical analysis of photospheric magnetic flux features over a full solar cycle using data collected by the Helioseismic and Magnetic Imager (HMI) instrument on the Solar Dynamics Observatory (SDO). The methods and data we use are very similar in general to Song et al. (2024). However, a major difference is that our study tests a number of different distributions against our data set (more details in Section 2) to determine the best fitting distribution over a full solar cycle.

In Section 2 we introduce the data set and our method of analysis before highlighting our results in Section 3. We discuss the implications of our findings in Section 4 and provide some concluding remarks in Section 5.

## Data

We use a sequence of 260 full disk line-of-sight magnetograms from the Helioseismic and Magnetic Imager (HMI) on the Solar Dynamics Observatory (SDO) taken at midnight on the 1st and 16th day of each month from 01 May 2010 to 16 March 2021. The dates were chosen arbitrarily but they provide at least half a Carrington rotation (27.27 days) between subsequent observations meaning that for each month we get two completely independent views of the photosphere. Three exceptions to these dates are 16 September 2010, 16 December 2011 and 16 March 2021 where we take the map at 00:12:00 the same day, 23:12:00 the day before and 00:12:00 the same day, respectively. This is due to a lack of good data at midnight of those days. This gives us enough data to span almost a full solar cycle. Magnetograms measure the magnetic flux density (in Mx cm^−2^) on the solar photosphere, however we use the magnetic flux density as a proxy for the average magnetic field strength at each pixel (measured in Gauss, G). Each magnetogram is a 4096 × 4096 pixel image with a pixel area of 0.5 × 0.5 arcsec^2^. The data is corrected from line-of-sight to radial magnetic field strength using a radial cosine correction. Each pixel is multiplied by the area of the Sun which it represents in order to convert the data into a measurement of radial magnetic flux. To avoid inaccuracies in radial magnetic flux near the limb we ignore all pixels further than $60^{\circ}$ in latitude or longitude from the center of the Sun.

### Feature Identification Method

#### Comparison of Different Methods

**Figure 1:**
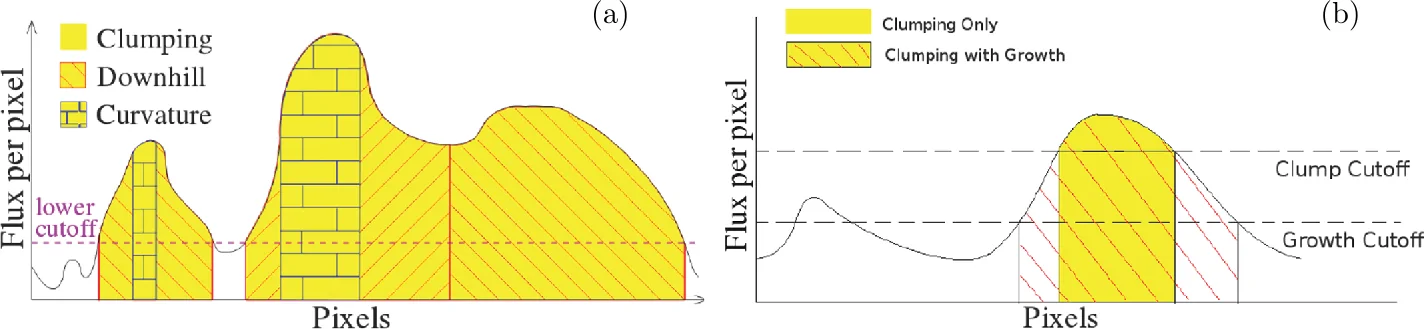
(a) A cartoon illustration of three common feature identification algorithms. The *clumping* method, as originally devised by Parnell (2002), uses a single lower cut-off and identifies two separate features. The *downhill* method splits the larger feature into two separate features at the local minimum (in 3D this would be a saddle point). The *curvature* method only identifies two small features; the peak at the right hand side is too flat for the method to identify. (b) A cartoon illustration of the filtering process carried out by the separate thresholds. The left hand peak is created by ‘noisy’ pixels and should therefore be neglected. The *clump* cutoff, which is the same as the lower cut-off in panel (a), achieves this, however, it removes some pixels which we believe are genuine constituents of the feature at the right hand side. Expanding the feature out to the *growth* cutoff allows us to obtain better estimates of what we believe to be the feature’s true size.

In order to identify and isolate individual magnetic features in a magnetogram we use a modified version of the *clumping* algorithm developed by Parnell (2002). The *clumping* algorithm defines a magnetic feature as a “contiguous group of same-signed pixels with absolute values greater than a lower cutoff”. These groups of pixels are known as ‘flux massifs’ as they are analogous to a mountain massif. There are two other common feature detection algorithms (DeForest et al. 2007), both of which have different definitions of magnetic features. The first of which, known as the *downhill* method (Welsch and Longcope 2003), finds flux peaks rather than massifs. The algorithm divides massifs into their individual ‘summits’ along saddle lines. The last method, the *curvature* method (Strous 1994; Hagenaar et al. 1999), finds flux cores by identifying local extrema and grouping together contiguous pixels that form a convex surface around the extrema. Figure 1 (a) shows a cartoon illustration of the differences in identification of features by the three different methods. We decided to employ the *clumping* algorithm as we feel it is the most robust to changes in the resolution of data being analysed. We decided against the *downhill* and *curvature* methods as they are sensitive to the number of local extrema. Increasing resolution tends to increase the number of individual ‘peaks’ within a flux massif and, hence, the *downhill* and *curvature* methods will identify many smaller features rather than the flux massif itself. We believe the flux massifs are the best representation of individual magnetic features. The *clumping* algorithm holds up well to increases in resolution as it does not split up the features present in lower resolution data. Therefore, any newly identified features would be a result of the increased resolution only. In addition, the *curvature* method is disregarded as it also fails to identify any features that have a ‘flat’ top due to their lack of curvature as is shown in Figure 1. This may be an uncommon occurrence but is worth taking into account.

#### Modification of Clumping Algorithm

We have modified the *clumping* algorithm by allowing for a second threshold (known as the *growth* cutoff) which is lower than the original threshold (known as the *clump* cutoff). The *growth* cutoff allows us to expand the identified features by including any bordering pixels that have an absolute value greater than the *growth* cutoff. We do this so we can identify features more accurately by including all pixels which contribute to the feature and avoid any ‘false positive’ features. We set the *clump* cutoff high enough to avoid identifying ‘noisy’ pixels as features but in doing so we omit any contributing pixels which are below the threshold. The *growth* cutoff allows us to incorporate these pixels into their respective features. The two threshold modification can alternatively be viewed as the *growth* cutoff acting as the original threshold which needs to be satisfied and the *clump* cutoff acting as a filter which tries to remove any ‘false positives’. Figure 1 (b) shows a cartoon illustration of this filtering in practise. As a final step we neglect all identified features which do not consist of at least five contiguous pixels.

#### Choice of Thresholds

**Figure 2:**
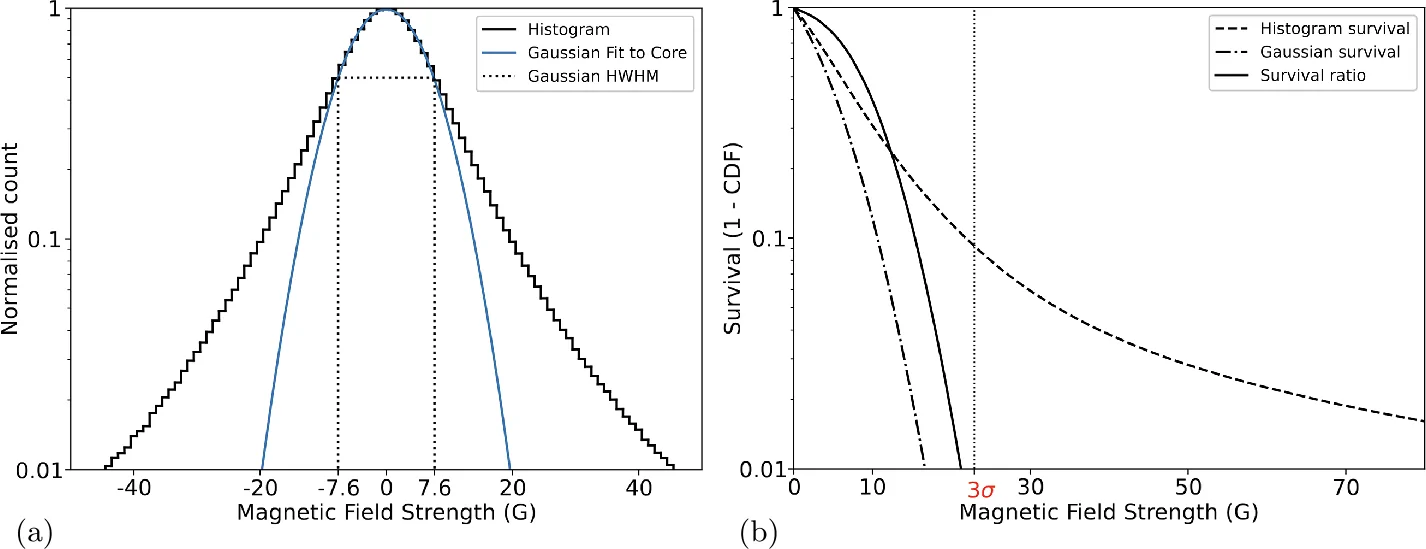
(a) A histogram (black solid line) of the magnetic field strength of the pixels is created. A Gaussian curve (blue solid) is fitted to the core of the histogram (any bins with count greater than or equal to 0.5). The full width at half maximum (FWHM) is also plotted (dotted). The noise of the magnetogram is estimated as the half width at half maximum (HWHM). For this magnetogram the noise level is approximately 7.6 G. (b) The survival function of the histogram (dashed) and the Gaussian curve (dash-dotted) are plotted. The ratio of the two curves (solid) indicates the fraction of the pixels above a certain magnetic field strength that are associated with noise. From the graph we observe that for a threshold of 3 times the noise level (22.8 G), less than 1% of the pixels above the threshold are associated with noise. This allows us to comfortably set a *clump* threshold of 3 times the noise level and a growth threshold of 2 times the noise level to better estimate the true size of the features.

The two cutoff values are applied as a means to neglect noisy pixels; therefore, we use the noise levels of the magnetograms themselves to assign the cutoff values. We estimate the noise level of the magnetograms following the technique of Parnell (2002). We fit a Gaussian curve to the core of a histogram of the pixel values assuming that the core of the histogram is associated with the noisy pixels. The range of the noisy pixels is then given as the full-width at half-maximum (FWHM) of the Gaussian fit and the noise level assumed to be the half-width at half-maximum (this accounts for the sign of the individual pixels). To give an illustration of the method, Figure 2 (a) shows the histogram of the magnetogram taken on 01 May 2014 with the Gaussian fit superimposed. Also shown is the FWHM (dotted). In this case the noise level is approximately 7.6 G. To determine the thresholds we perform a survival analysis; a statistical technique which can be used to determine the proportion of a population which will survive past a certain time. In our case we can use survival analysis to determine the fraction of pixels which are associated with noise. Figure 2 (b) shows the survival function for both the histogram and the fitted Gaussian (dashed and dash-dotted, respectively). The survival function is defined as 1−F(x), where F(x) is the cumulative distribution function for the observed distribution. The ratio of the survival functions of both curves (solid) tells us what fraction of pixels with field strength above a certain value are associated with noise. From the figure we see that for pixels with magnetic field strength greater than 3 times the noise of the magnetogram (22.8 G) less than 1% of these are associated with noise. We therefore choose the *clump* threshold to be 3 times the noise level and allow for growth of features back to a *growth* threshold set at 2 times the noise level. However, the noise level is different for every magnetogram and we want to ensure uniformity of feature identification across the full solar cycle.

**Figure 3:**
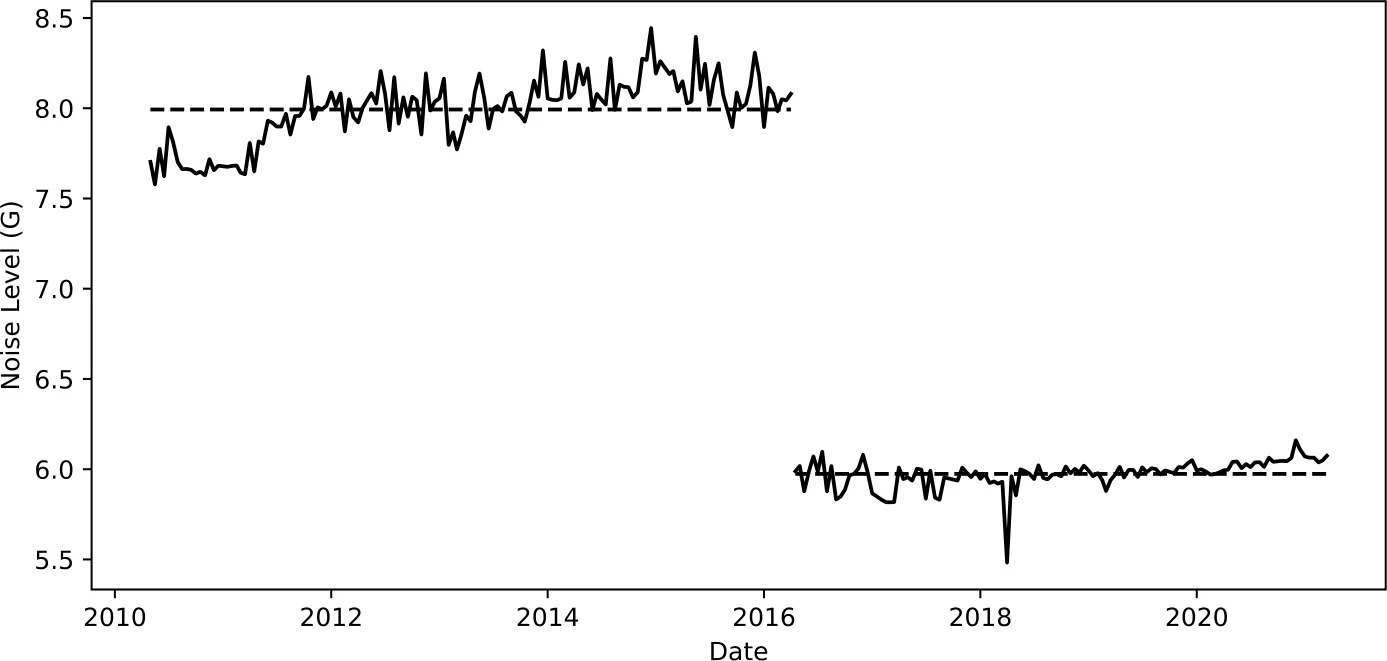
The noise level across the full data set. The drop in noise level is caused by a change in the way HMI takes measurements (Liu et al. 2016) which reduced the noise associated with magnetograms. The dashed lines show the average value of the noise level before and after the drop; approximately 7.993 G and 5.974 G, respectively. There is no obvious correlation between noise level and solar cycle so we use the average noise values as the thresholds for all maps to ensure uniformity in the feature detection.

Figure 3 shows the value of noise for each magnetogram in our data set. The first thing to note is the significant drop in noise level during April 2016. This is caused by a change in HMI’s observing scheme, known as “Mod-L”, to now combine measurements from two separate cameras. The result of this change is that the noise of magnetograms from HMI is significantly reduced (see Liu et al. 2016, for more details). The average values of the noise before and after the switch are approximately 7.993 G and 5.974 G, respectively. There is no significant change in the noise levels over time, therefore we can assume that using the average noise value before and after the drop as a proxy for the true noise is acceptable and provides uniformity across all maps. We then calculate the two thresholds, *clump* and *growth*, by multiplying the average noise level by 3 and 2, respectively. We apply these thresholds to the modified clumping method outlined in Section 2.1.2 and determine all magnetic features present on each magnetogram.

## Results

**Figure 4:**
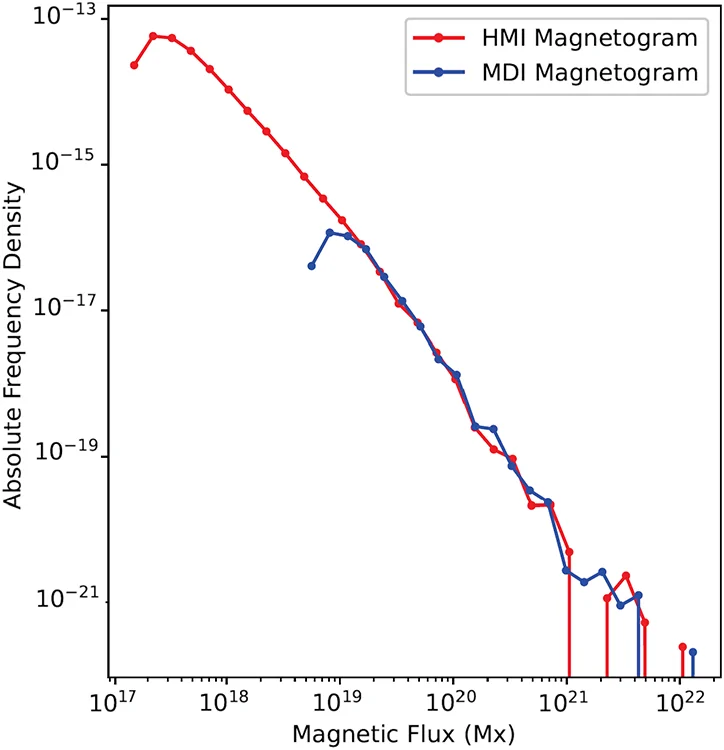
Histogram of frequency density of magnetic flux features detected in SDO HMI magnetogram taken at midnight on 16 March 2011 (red) and detected in a SOHO MDI magnetogram taken at approximately the same time (blue).

Following the example of previous work (e.g. Parnell et al. 2009), we use histograms to analyse the observed distribution of magnetic flux features. Figure 4 is an example for such a histogram. It shows the absolute frequency density of magnetic flux for magnetogram data taken on 16 March 2011, plotted on double-logarithmic axes. This example histogram highlights some general properties of magnetic flux histograms.

Firstly, over a considerable range of magnetic flux ($10^{18}\,\mathrm{--}\,10^{20}~\mathrm{Mx}$) the distribution seems to be well approximated by a straight line. In a double-logarithmic plot this is interpreted as an indicator of a power law distribution (e.g. Newman 2005; Parnell et al. 2009, see the Appendix for mathematical details).

Secondly, however, one notices that above $10^{20}~\mathrm{Mx}$ the data becomes sparser and the straight-line behaviour is less evident. It is one of the aims of this paper to investigate the nature of the magnetic flux distribution over the full range of flux values; another aim is to find out whether and how the distribution varies over a solar cycle.

Thirdly, we notice a turnover in the lower flux tail ($< 10^{18}~\mathrm{Mx}$). For context we show in Figure 4 a comparison between the SDO HMI data and a SOHO MDI observation taken at the same time (for details how to account for the difference in measurement between MDI and HMI, see Liu et al. 2016). As one can see in Figure 4 the MDI based histogram has a turnover at a higher magnetic flux value due to the lower resolution of MDI compared to HMI, while otherwise the histograms seem to match closely. Although this is not a proof, we take this as an indication that the turnover in the HMI histograms is also caused by the limitation in the observational resolution of the instrument.

As the drop-off seems to be caused by instrumental effects, we have to exclude it if we are to obtain the true underlying distribution of magnetic flux features. To account for this we truncate the dataset to only include features with a magnetic flux greater than $10^{18}~\mathrm{Mx}$ and re-bin the histogram. This limit is chosen somewhat arbitrarily but setting a hard limit is a more sensible approach than trying to determine separate limits on a case-by-case basis.

**Figure 5:**
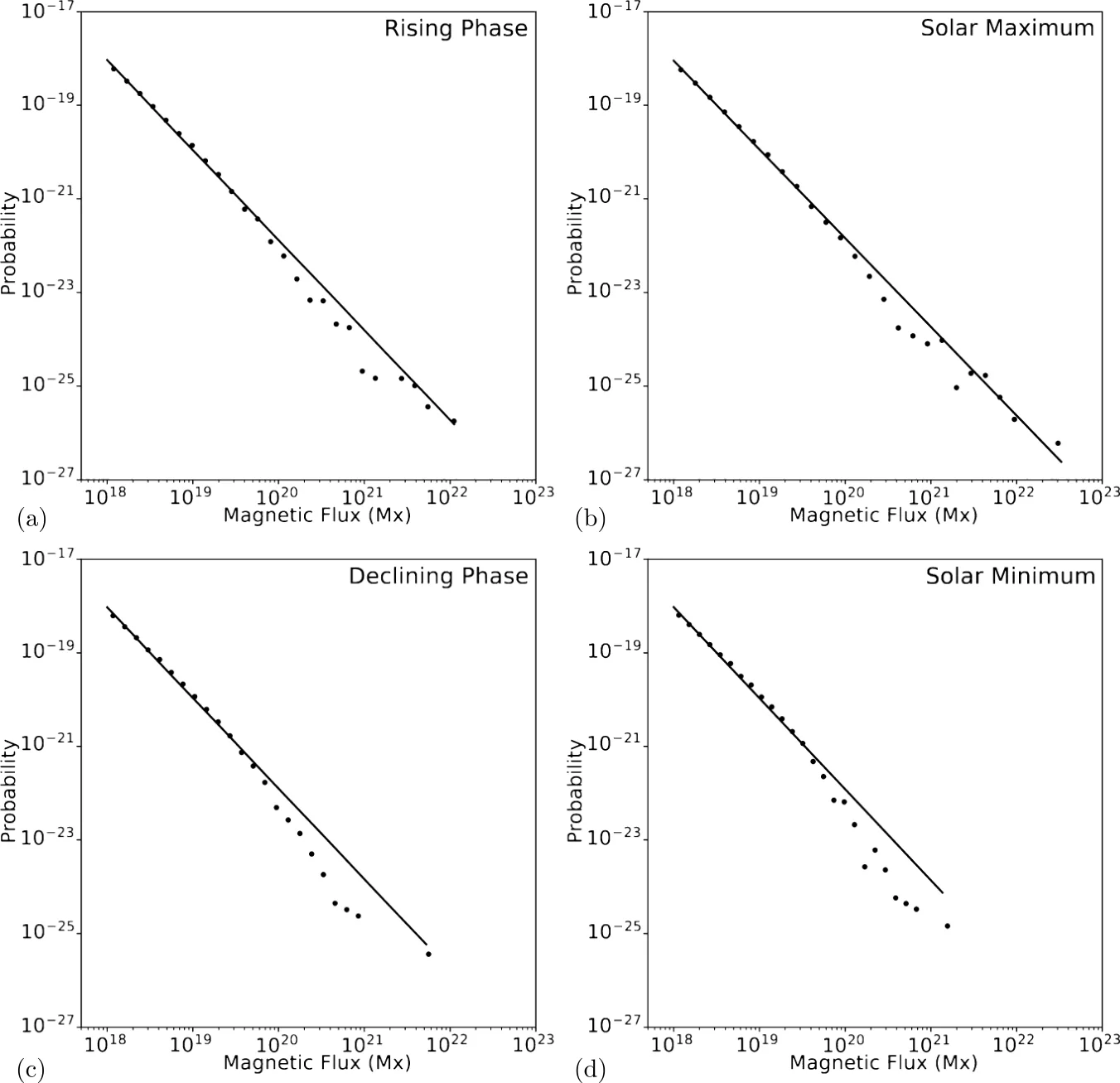
Histogram of magnetic flux features and a fitted power law model at different times in the solar cycle. (a) 16 March 2011 representing rising activity. (b) 01 July 2014 representing solar maximum. (c) 16 August 2017 representing declining activity. (d) 16 August 2020 representing solar minimum.

As already mentioned before, one notices that the truncated histogram data resemble a straight line in a double-logarithmic plot. This is usually interpreted as being indicative of an underlying power distribution function (e.g. Newman 2005; Parnell et al. 2009). Figure 5 shows histograms of the observed distribution of magnetic flux at different phases of the solar cycle, together with a power law model that has been fitted to the data using the maximum likelihood method (e.g. Scholz 2014).

One can see that the power law fits the data reasonably well in each case. However, it is also clear that in the higher flux tail the power law model starts to deviate from the observed distribution. The histogram values tend to be lower than the power law model; this is especially true during the declining phase and solar minimum. Therefore the question arises whether a power law is really the optimal mathematical model to represent the data or whether other distribution functions might represent the data better.

We have picked a (single) power law based on a purely subjective interpretation of the data. Clearly, more sophisticated means are needed to make an objective decision on whether or not a mathematical model is an accurate representation of the true distribution that the data come from. In this paper we use a number of different methods to achieve this.

We also want to investigate whether there might be alternative models which perform better than the single power law over the full solar cycle. Therefore, in addition to the single power law, we will test eight different probability density functions (PDFs) against the data:

- Exponential PDF
- Lognormal PDF
- Weibull PDF
- Truncated Weibull PDF (abbreviated as *Tr. Weibull* from now on)
- Sharp Double Power Law PDF (abbreviated as *Sharp-DPL* from now on)
- Smooth Double Power Law PDF (abbreviated as *Smooth-DPL* from now on)
- Weibull-Lognormal PDF
- Truncated Weibull-Lognormal PDF (abbreviated as *Tr. Weibull-Lognormal* from now on)

The mathematical details for each of these distributions can be found in the Appendix. We remark that apart from the single power law a few more of these distribution functions (exponential, lognormal, and Weibull) have been discussed in the context of magnetic flux distributions before (see e.g. Muñoz-Jaramillo et al. 2015).

**Figure 6:**
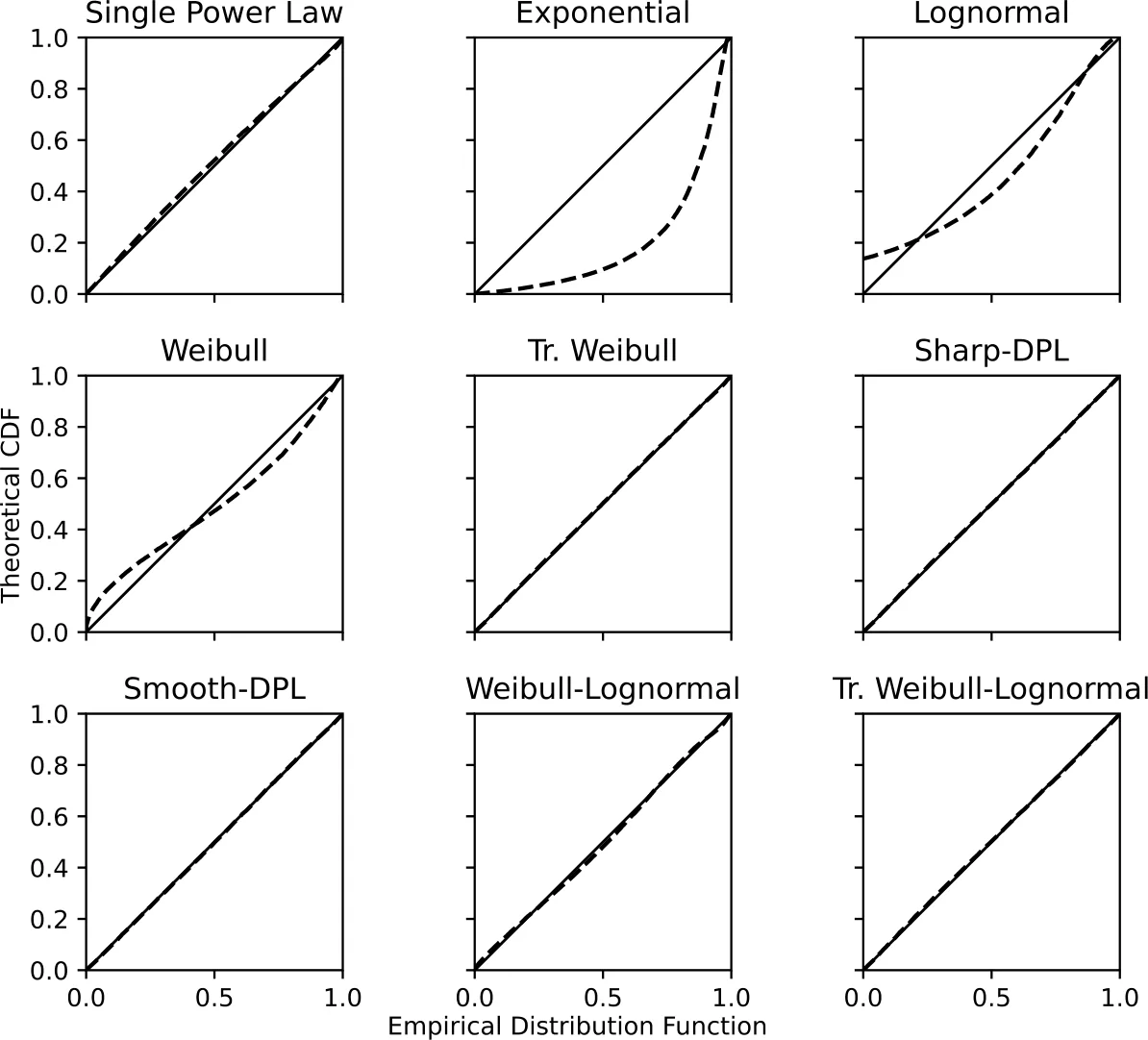
Example P-P plots for the nine model PDFs considered in the paper. These plots use data from 1 July 2014, which is around solar maximum. The solid line in each plot is the main diagonal and the dashed line is the curve generated by plotting the theoretical CDF against the empiral distribution function. See main text for more details.

We start our investigation of these model PDFs by first using a better graphical representation to assess how well a model distribution function fits the data called probability-probability plots (P-P plot). A P-P plot displays a model’s cumulative distribution function (CDF) against the empirical cumulative distribution function (e.g. Gibbons and Chakraborti 2020) on the unit square. A good fit between the model CDF and the empirical CDF would lead to a curve very close to the main diagonal. A typical example of P-P plots for all nine model distribution functions is shown in Figure 6 for a date around solar maximum (1 July 2014). From Figure 6 it is clear that the exponential, the lognormal, and the Weibull distribution do not seem to represent the data as well as the other six distribution functions. However, although P-P plots give a better impression of the quality of fit for a model probability density function, the results are still subjective. Furthermore, applying this method to all datasets in our sample would not be practical.

Clauset, Shalizi, and Newman (2009) suggest that one way to check if a model is suitable or not is to determine the model’s ‘plausibility’ by generating a p-value based on some goodness-of-fit statistic. We follow their approach by generating the so-called p-value based on the Kolmogorov-Smirnov statistic (KS Stat). The KS Stat is a measure of the maximum difference between a model’s cumulative distribution function and the observed data’s empirical distribution function (e.g. Chakravarti, Laha, and Roy 1967).

The p-value is determined by fitting a model to the data and calculating the KS Stat for the model. Synthetic datasets are then generated based upon the fitted model’s parameters, before refitting the model to the synthetic dataset and calculating a new KS Stat. The p-value is the fraction of data sets that have a larger KS Stat than the original data. Again, following the example of Clauset, Shalizi, and Newman (2009), a model is rejected as implausible if the p-value is below 0.1.

In Table 1 we provide the p-values for all nine model probability density functions for the same data used in the P-P plots in Figure 6. One can see that the p-values for the Exponential, Lognormal, and Weibull distributions are zero and hence below the threshold values of 0.1, which corroborates the conclusion that these three PDFs do not represent the data well and should be rejected.

**Table 1:** p-values for the plausibility of all nine models for 1 July 2014. Models which surpass the 0.1 significance level are determined to be plausible fits to the data and are highlighted in bold.

Distribution	*p*-value
**Single Power Law**	**0.4**
Exponential	0
Lognormal	0
Weibull	0
**Tr. Weibull**	**1**
**Sharp-DPL**	**1**
**Smooth-DPL**	**1**
**Weibull-Lognormal**	**0.2**
**Tr. Weibull-Lognormal**	**1**

Obviously, one should not base the rejection of a probability density function on a comparison with a single data set. Therefore we have calculated the p-values for all models over the complete data set covering the full solar cycle.

It turns out that the p-values of the Exponential, Lognormal and Weibull PDFs are very close to zero throughout the complete data set. Plots of the p-values for these PDF models against time do not convey any additional information and are hence omitted. On the basis of this result we will discard these three PDF models from the analysis.

**Figure 7:**
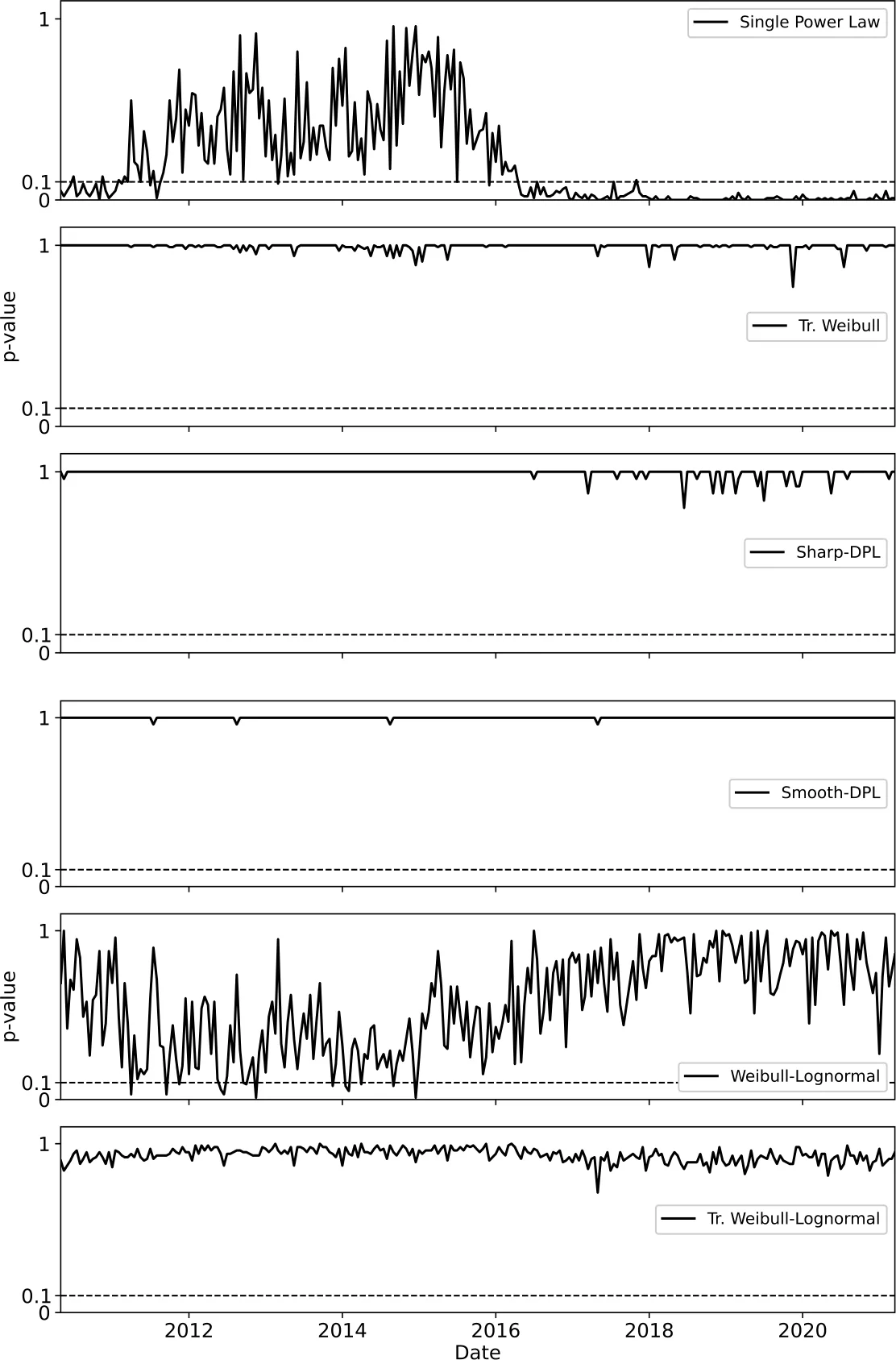
Variation of p-values for the remaining six PDF models over the full solar cycle. The dashed line shows the 0.1 significance level which plausible models must exceed.

Figure 7 shows the temporal variation over the solar cycle of the p-value for each of the remaining six models. In each plot, the 0.1 significance level is shown as a dashed line. For plausibility, a model’s p-value must exceed 0.1.

For the Truncated Weibull, Sharp Double Power Law, Smooth Double Power Law and Truncated Weibull-Lognormal model PDFs, the p-value is safely above the 0.1 threshold for all magnetograms in the sequence. We conclude that each of these models are plausible fits to the magnetic flux distribution at all times in the cycle.

However, both the Single Power Law and Weibull-Lognormal models drop below 0.1 on occasion. Interestingly, the Single Power Law’s p-value shows correlation with the solar cycle, increasing as magnetic activity increases, and the Weibull-Lognormal model appears to be anti-correlated, decreasing as magnetic activity increases. This suggests that the Single Power Law may be a good fit at solar maximum but fails at solar minimum and likewise, the Weibull-Lognormal may be a good fit at solar maximum but may fail at solar minimum.

We therefore omit both the single power law and the Weibull-Lognormal PDFs from the further analysis and accept the other four PDFs as plausible fits to the data.

To be able to make a choice which of the remaining four PDF models is the most suitable, we compare a number of different goodness-of-fit tests across the full solar cycle. We have based our choice of goodness-of-fit test statistics on those employed by Muñoz-Jaramillo et al. (2015); namely the KS Stat, the relative Akaike Information Criterion (Rel. AIC), and the Akaike weights ($A_{w}$). However, we have added a fourth test which measures the relative log-likelihood of each model (Rel. Log-Lik).

The goodness-of-fit statistics are summarised in Tables 2 and 3 which show the average value, over the full solar cycle, of each test statistic for all four models, and the percentage of time that each model wins a particular goodness-of-fit comparison, respectively. It is clear from these tables that the smooth double power law is the best performing model for most of the solar cycle. It is difficult to decide whether the sharp double power law PDF or the truncated Weibull-lognormal PDF follow in second place based on the win percentages alone, but the truncated Weibull-lognormal PDF comes out ahead on average values. Finally, it is clear that the truncated Weibull PDF is the worst performer over the full cycle.

**Table 2:** The average value, over the full solar cycle, of the four goodness-of-fit statistics for each model.

Model	Average value			
	KS Stat	Rel. AIC	$A_{w}$	Rel. Log-Lik.
Tr. Weibull	0.0073	19.532	0.036	−11.804
Tr. Weibull-Lognormal	0.0062	7.469	0.176	−2.772
Sharp-DPL	0.0082	10.497	0.211	−6.286
Smooth-DPL	0.0055	1.966	0.577	−1.020

**Table 3:** The amount of time each model wins the comparison of each goodness-of-fit statistic, given as a percentage of all magnetograms.

Model	Win percentage (%)			
	KS Stat	Rel. AIC	$A_{w}$	Rel. Log-Lik.
Tr. Weibull	20.00	2.69	2.69	0.00
Tr. Weibull-Lognormal	25.77	13.07	13.07	26.54
Sharp-DPL	6.54	21.92	21.92	6.92
Smooth-DPL	47.69	62.31	62.31	66.54

Although the smooth double power law does not win all goodness-of-fit comparisons 100% of the time, it seems sensible to select the model which performs best on average. All four models are plausible fits to the data, but we wish to make a definitive choice in order to perform statistical inference. Inferences about the data may come in a number of different forms, for example, using the model to make future predictions of the long-term trends in the data; to provide a physical constraint in numerical models of the sub-surface processes; or to achieve a better theoretical understanding of the underlying solar dynamo processes.

**Figure 8:**
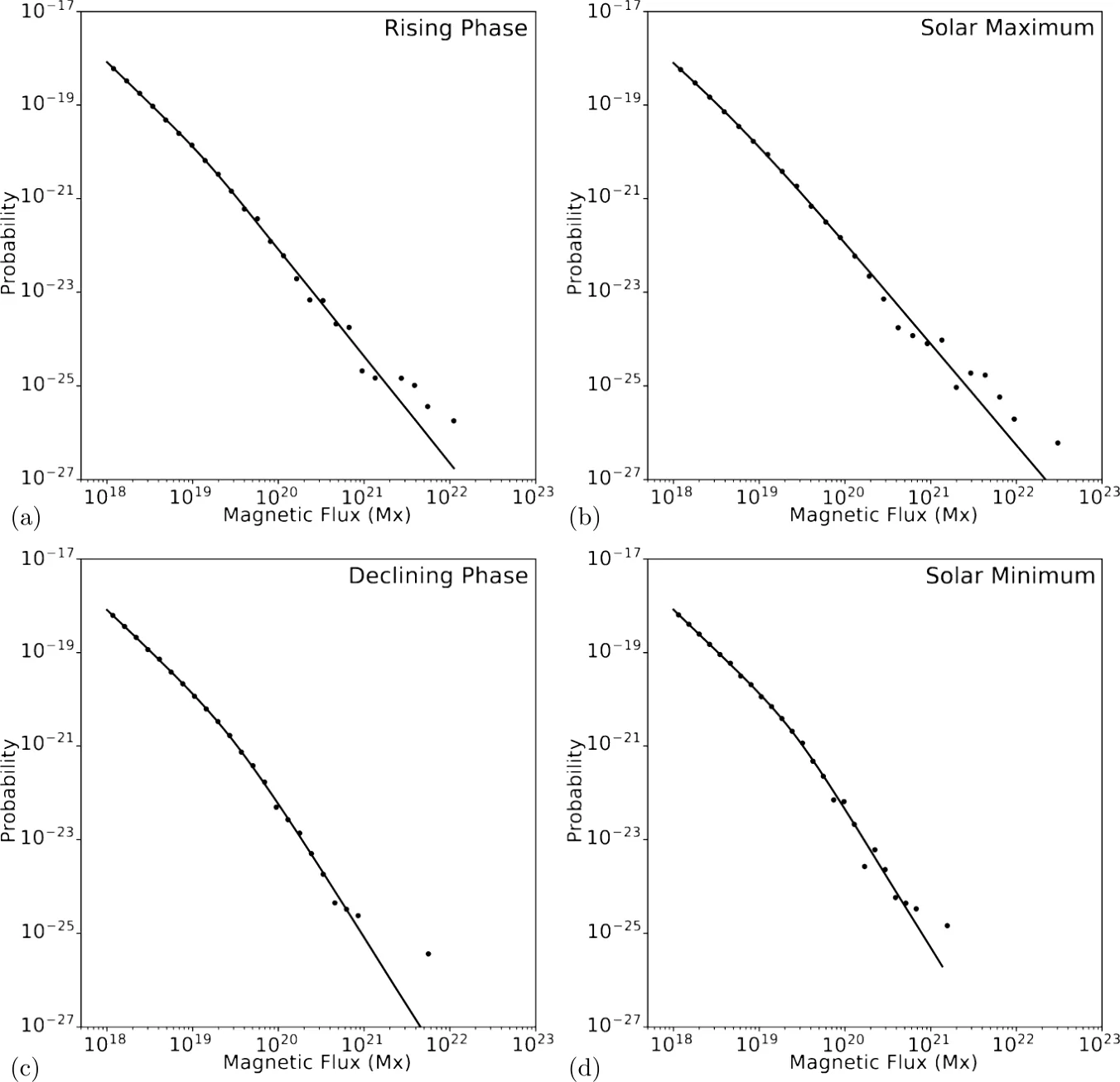
Histograms of the same data as in Figure 5 but with a smooth double power law model fitted to the data rather than single power law model.

Therefore we choose the smooth double power law as the best representation of the true distribution of magnetic flux features over the course of a full solar cycle. Figure 8 shows the histograms of magnetic flux distribution representing the same four phases of the solar cycle as in Figure 5 but with a smooth double power law model overplotted on the histogram rather than a single power law model. The smooth double power law’s flexibility allows it to capture the behaviour at the high flux tail to a much higher accuracy than the single power law. We remark that this result is largely in accordance with the “two-segment” power law distribution put forward by Song et al. (2024), although their distribution seems to correspond to what we call sharp double power law in this paper.

## Double Power Law: Variation over One Solar Cycle

**Figure 9:**
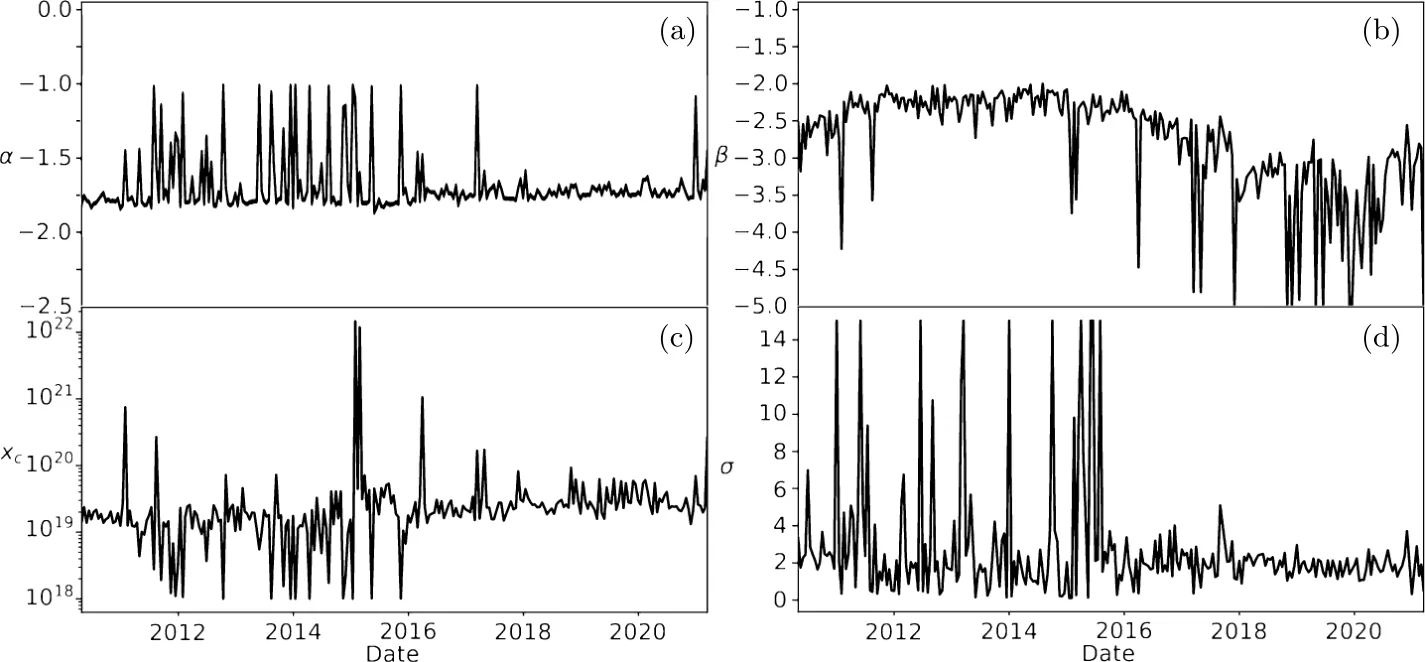
Temporal variation of the parameters of the smooth double power law model over the solar cycle. Panel (a) shows α, panel (b) shows β, panel (c) shows $x_{c}$, and panel (d) shows σ.

A more in depth understanding of the double power law model can be obtained by investigating the temporal variation of the slope parameters of the separate power laws. Figure 9 shows how the fitted parameters of the smooth double power law model vary over one solar cycle.

Panel (a) shows the temporal variation of α, the slope of the left hand power law. Typically, the value of α lies between −1.7 and −1.8 but there are plenty of upward fluctuations in the curve. There is much more consistency in the value at times of reduced solar activity (from 2017 onwards reaching solar minimum in late 2019). Although the fluctuations might simply be a reflection of statistical variations in the data, one other possible explanation could be that the decay of large scale active regions does contribute to the small scale flux distribution over shorter time scales. This would explain why these fluctuations seem to occur mainly during enhanced solar activity.

Panel (b) shows the temporal variation of β, the slope of the right hand power law. There is a noticeable trend: the slope (magnitude of β) becomes steeper at solar minimum and flatter at solar maximum. Again, there are a number of outliers in the graph. Interestingly, the spikes all point towards more negative values, whereas the spikes in the α curve all point towards less negative values. A value of −5 was set as the lower limit in the maximum likelihood estimation optimisation algorithm, so it is possible that the true value of β in some magnetograms is less than −5. However, due to numerical limitation we have to compromise slightly on accuracy. For the most part, the value of β is greater than −5. The upper envelope of the curve seems to vary roughly between −3.0 (low activity) and −2.0 (high activity)

The temporal variation of the ‘kink location’ ($x_{c}$) is shown in panel (c). Once again, the data is spiky but typically the value lies between $10^{19}$ and $10^{20}~\mathrm{Mx}$. The values seem to be slightly higher at solar minimum than at solar maximum but whether this is a real feature of the distribution or the result of statistical fluctuations is unclear. This question could be investigated further in the future.

Finally, in panel (d) we see the temporal variation of the smoothing parameter (σ). A larger value of σ means the transition from slope α to slope β is sharper. Low values of σ means that the transition takes place over a wider range. Typically, the value lies between 2 and 4, however there are a number of spikes in either direction (both toward a value of 0 and toward a value of 15). A value of 15 was set as an upper limit for the maximum likelihood estimation optimisation algorithm. The spikes toward the highest value of σ could be even higher, but we argue that a value of 15 is already sharp enough to convey the required information. In addition, computational limitations, again, mean we have to make a compromise on accuracy in these situations.

We see that there is much more variation in the β-parameter than the α-parameter. This tells us that the slope of the small-scale flux distribution is much more stable over a solar cycle than that of the large-scale flux distribution. The right hand slope (β) varies with changing solar activity; typically having a shallower slope at solar maximum and a steeper drop-off at solar minimum. This is to be expected because the right hand power law slope (β) is affected by the presence of large-scale active regions.

The fact that the power law index of the small-scale flux distribution appears to remain unchanged on average regardless of the level of solar activity could be interpreted as an indication that the long-term behaviour of the small-scale flux distribution is not dominated by the fragmentation of larger features, but largely due to a mechanism which perpetually generates new, small-scale features throughout the entire solar cycle.

We note that our results are qualitatively consistent with those of Song et al. (2024). Although we did not assume from the outset that a double power law distribution is the correct fit for the data, we found using statistical goodness of fit tests that over a full solar cycle a smooth double power law is the best fitting distribution function amongst those we tested. This very similar to the “two-segment” power law distribution fitted by Song et al. (2024), although this would correspond to our sharp double power law and not to the smooth double power law we found to be the best fit over the full solar cycle. Quantitatively, we found that our power law index for the small scale flux part (α) is roughly between −1.7 and −1.8 with more upward fluctuations during solar maximum, whereas Song et al. (2024) quote a slightly larger average value of −1.64. We note that it is possible that when averaging over the whole cycle we would arrive at a higher value due to the upward fluctuations we find. However, given the nature of the variations of α as shown in panel (a) of Figure 9 we do not believe that an average value would necessarily be meaningful. Our value of the power law index for the large scale flux part (β) is roughly in the same range, but at some times considerably lower than the corresponding values given by Song et al. (2024), which vary between −1.92 at solar maximum and −2.27 at solar minimum. We would suggest that this difference could be due to the difference between the sharp double power law (“two-segment” power law) used by Song et al. (2024) and the smooth double power law which we use. This is also reflected by the fact that Song et al. (2024) fix the transition point between the two segments of their double power law to $5.5 \times 10^{18}~\mathrm{Mx}$, whereas we have a transition region whose position ($x_{c}$) and width (σ) is part of the fitting process. Despite these differences which we consider to be relatively minor, we find it very encouraging that our findings using a statistical goodness of fit approach seem to give results that are largely consistent with the findings by Song et al. (2024).

## Summary and Conclusions

In this paper we have presented the analysis of 260 full disk line-of-sight HMI magnetograms over an 11 year period from May 2010 to March 2021. We used the modified clumping algorithm to identify individual magnetic flux features and generated size distributions of these features. We then used the maximum likelihood method to obtain fits to these observational size distributions by nine different probablity density functions: (single) power law PDF, expontial PDF, lognormal PDF, Weibull PDF, truncated Weibull PDF, sharp double power law PDF, smooth double power law PDF, Weibull-lognormal PDF, and truncated Weibull-lognormal PDF.

Using a variety of statistical goodness-of-fit criteria we established that over a full solar cycle the smooth double power law seems to represent the best fit to the observations. We analysed how the parameters of the smooth double power law change over the solar cycle. On the basis of this analysis we found that the power law index for the small flux features (α) shows no long-term solar cycle variation, whereas the power law index for the large flux features (β) shows a variation which is in line with the decrease of large scale active region flux during solar minimum. The two other parameters of the smooth double power law PDF are the location of the transition between the two power laws ($x_{c}$) and the width of the transition (σ). On the basis of our analysis we could not identify any significant long-term variation of these two parameters over the full solar cycle.

The smooth double power law is an example of a bi-modal distribution. Bi-modal distributions are often taken to be an indication that the two different parts of the distribution could be generated by different physical processes. If interpreted in such a way our result could be viewed as being supportive of processes keeping the small-scale flux distribution largely time-invariant over a solar cycle, for example small-scale dynamo processes as discussed in Rempel et al. (2023), while the solar cycle variation of the large-scale flux distribution is generated by a large-scale dynamo. However, one has to be cautious not to overinterpret our results for a number of reasons. Firstly, the analysis presented in this paper is based on a single solar cycle only. It will need to be confirmed over other solar cycles, which clearly is a long-term project. Secondly, our results do not exclude the possibility that a single physical mechanism could be responsible for both the relative temporal stability of the flux distribution at small scales while also generating the variation of the large-scale distribution over the solar cycle. A solid theoretical understanding of the various physical processes is necessary to be able to distinguish between the different options. Finally, we note that the power indices as well as the other parameters of the flux distribution are subject to statistical fluctuations. While the overall long-term trend in the (large-scale) power law index β is relatively obvious, we cannot exclude the possibilty that the statistical fluctuations could prevent us from detecting much smaller systematic variations in α, for example. In this context, we mention the work by Korpi-Lagg et al. (2022) who found no signficant solar cycle dependence of the quiet sun internetwork magnetic field fluctuations when using $1^{\circ}$ data patches, but clear solar cycle dependence of network and internetwork magnetic field fluctuations when using larger patches ($15^{ \circ}$). Although the paper by Korpi-Lagg et al. (2022) studies a different quantity than we did in the current paper, it shows that the question of solar cycle dependence of small scale magnetism is a subtle one. Clearly, further studies of this important problem are warranted, both on the observational/data analysis side and on the theoretical side.

## Figures and Tables

**Figure 10:**
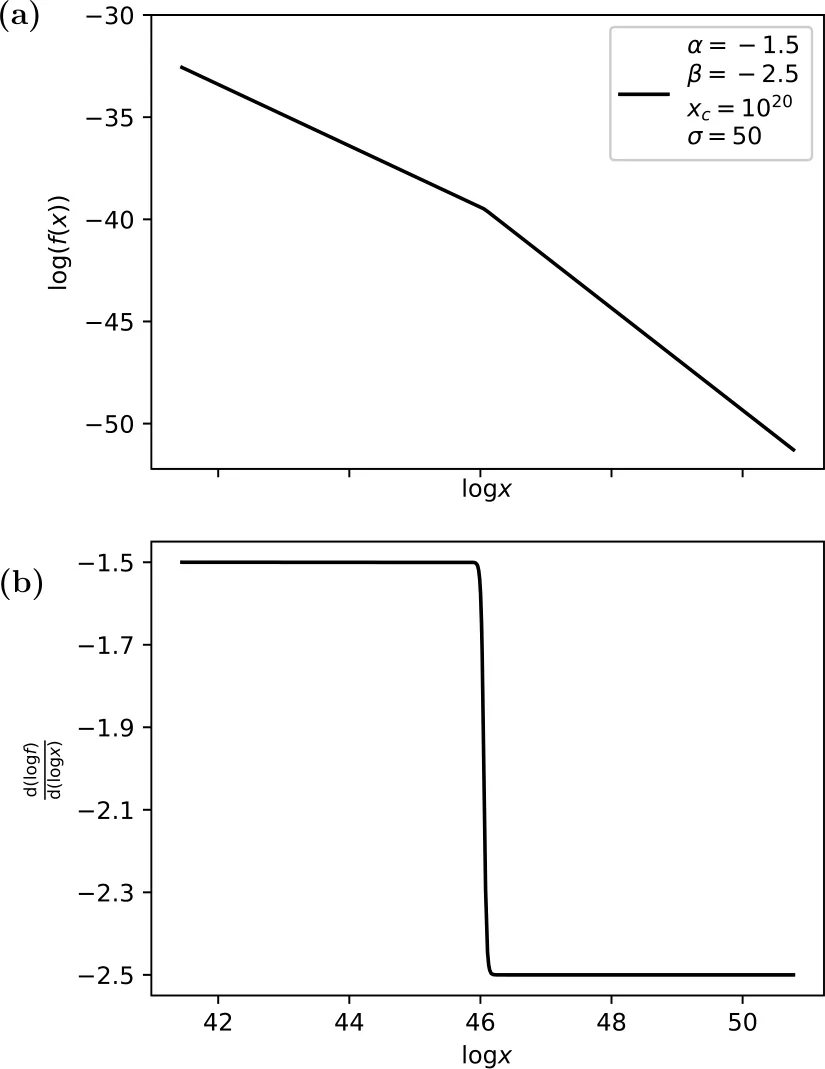
(a) A typical double power law will have the same structure as shown in the graph, constrained by four parameters; on a log-log scale the function will display two different straight line behaviours. The lines represent different power laws which transition from one to the other at some location, $x_{c}$, which we call the ‘kink’ location. To the left of the kink we have a power law with index α and to the right we have a power law with index β. The rate at which the function transitions from one power law to the other is controlled by a fourth parameter, σ; larger values of σ lead to a quicker transition and hence a sharper kink. (b) The derivative of the function (in log-log space) allows us to mathematically formulate the double power law function. We model the derivative as a hyperbolic tangent function shifted in the y-axis which is constrained by the four parameters; α and β which control the amplitude and shift in y-axis, $x_{c}$ which controls the location, and σ which controls the width of the function.

## Data Availability

The data used in this paper are publicly available at http://jsoc.stanford.edu/.

## Appendix: Distribution Functions

For each of the different distribution functions (excluding the double power law distribution) investigated in Section 3, we provide, here in Sections A.1 to A.4, details of the probability density function (PDF, $f(x; \theta _{i})$), cumulative distribution function (CDF, $F(x; \theta _{i})$), and an outline of how the maximum likelihood estimates (MLEs, $\hat{\theta}_{i}$) of the parameters ($\theta _{i}$ for i=1,2,…,m; where m is the number of parameters) are determined. Section A.5 provides a more in-depth exploration of both forms of the double power law distribution; providing details of how the distribution is constructed and how the parameters may be estimated. It is important to note that the PDFs need to be multiplied by the total number of observed features in order to obtain the true frequency of fluxes.

### A.1 Single Power Law

The form of the normalised PDF defined over $x \in [x_{0}, \infty )$ for the single power law distribution is:

1 $$ f(x; \alpha ) = \frac{\alpha - 1}{x_{0}} \biggl(\frac{x}{x_{0}} \biggr)^{- \alpha}, $$

provided α>1. The CDF of the single power law is defined as:

2 $$ F(x; \alpha ) = 1 - \biggl(\frac{x}{x_{0}} \biggr)^{1-\alpha}. $$

The log-likelihood function for the single power law PDF is

3 $$ \mathcal{L}(\alpha ) = \sum_{i=1}^{N} \biggl[\ln{ (\alpha - 1 )} - \ln{x_{0}} - \alpha \ln{ \biggl( \frac{x_{i}}{x_{0}} \biggr)} \biggr], $$

where N is the total number of data points, $x_{i}$. Setting the derivative of Equation 3 with respect to α equal to zero and rearranging leads to the following formula for the MLE of α:

4 $$ \hat{\alpha} = 1 + \frac{N}{\sum_{i=1}^{N} \ln{ (\frac{x_{i}}{x_{0}} )}}. $$

### A.2 Weibull

The form of the normalised PDF defined over $x \in [x_{0}, \infty )$ for the Weibull distribution is:

5 $$ f(x; \beta , \gamma ) = \frac{\gamma}{\beta} \biggl( \frac{x - x_{0}}{\beta} \biggr)^{\gamma - 1}\exp{ \biggl(- \biggl( \frac{x - x_{0}}{\beta} \biggr)^{\gamma} \biggr)}, $$

provided $\beta , \gamma > 0$ The CDF of the Weibull distribution is defined as:

6 $$ F(x; \beta , \gamma ) = 1 - \exp{ \biggl(- \biggl( \frac{x - x_{0}}{\beta} \biggr)^{\gamma} \biggr)}. $$

The log-likelihood function for the Weibull PDF is:

7 $$ \mathcal{L}(\beta , \gamma ) = \sum _{i=1}^{N} \biggl[\ln{ \biggl( \frac{\gamma}{\beta} \biggr)} + (\gamma - 1 ) \ln{ \biggl( \frac{x_{i} - x_{0}}{\beta} \biggr)} - \biggl( \frac{x_{i} - x_{0}}{\beta} \biggr)^{\gamma} \biggr], $$

where N is the total number of data points, $x_{i}$. Setting the derivatives of Equation 7 with respect to both β and γ equal to zero and rearranging leads to the following formulae:

8 $$ \beta = \Biggl(\frac{1}{N}\sum _{i=1}^{N} (x_{i} - x_{0} )^{ \gamma} \Biggr)^{\frac{1}{\gamma}}, $$

9 $$ \frac{1}{\gamma} + \frac{1}{N}\sum _{i=1}^{N}\ln{ (x_{i} - x_{0} )} - \frac{\sum_{i=1}^{N} [ (x_{i} - x_{0} )^{\gamma}\ln{ (x_{i} - x_{0} )} ]}{\sum_{i=1}^{N} (x_{i} - x_{0} )^{\gamma}} = 0. $$

To obtain the MLE estimates for β and γ we first solve Equation 9 numerically to estimate $\hat{\gamma}$ and then determine $\hat{\beta}$ by substituting $\hat{\gamma}$ into Equation 8.

### A.3 Exponential

The form of the normalised PDF defined over $[x_{0}, \infty )$ for the exponential distribution is:

10 $$ f(x; \lambda ) = \lambda e^{-\lambda (x - x_{0} )}, $$

provided λ>0. The CDF of the exponential distribution is defined as:

11 $$ F(x; \lambda ) = 1 - e^{-\lambda (x - x_{0} )}. $$

The log-likelihood function for the exponential PDF is:

12 $$ \mathcal{L}(\lambda ) = N\ln{\lambda} - \lambda \sum _{i = 1}^{N} (x_{i} - x_{0} ), $$

where N is the total number of data points, $x_{i}$. Setting the derivative of Equation 12 with respect to λ equal to zero and rearranging leads to the following formula for the MLE of λ:

13 $$ \hat{\lambda} = \frac{N}{\sum_{i=1}^{N} (x_{i} - x_{0} )}. $$

### A.4 Log-Normal

The form of the normalised PDF defined over $[x_{0}, \infty )$ for the log-normal distribution is:

14 $$ f(x; \mu , \sigma ) = \frac{1}{x\sigma \sqrt{2\pi}}\exp{ \biggl(- \frac{ (\ln{x} - \mu )^{2}}{2\sigma ^{2}} \biggr)}, $$

provided σ>0. The CDF of the log-normal distribution is defined as:

15 $$ F(x; \mu , \sigma ) = \frac{1}{2} \biggl[1 - \mathrm{erf} \biggl( \frac{\ln{x} - \mu}{\sigma \sqrt{2}} \biggr) \biggr], $$

where $\mathrm{erf}$ is the error function. The log-likelihood function for the log-normal PDF is:

16 $$ \mathcal{L}(\mu , \sigma ) = -N\ln{\sigma} - N\ln{\sqrt{2\pi}} - \sum_{i=1}^{N}\ln{x_{i}} - \frac{\sum_{i=1}^{N} (\ln{x_{i}} - \mu )^{2}}{2\sigma ^{2}}, $$

where N is the total number of data points, $x_{i}$. Taking the derivative of Equation 16 with respect to both μ and σ leads to the following formula for the MLEs of μ and σ:

17 $$ \hat{\mu} = \frac{\sum_{i=1}^{N} \ln{x_{i}}}{N} $$

18 $$ \hat{\sigma} = \sqrt{ \frac{\sum_{i=1}^{N} (\ln{x_{i}} - \hat{\mu} )^{2}}{N} }. $$

### A.5 Double Power Law

The double power law distribution describes data which follow separate power laws in separate sections of the distribution’s domain, i.e. two separate power laws with different indices, α and β, describing two separate sections of the data, respectively. The location at which the function transitions from one power law form to the other, known as the ‘kink’ location, is denoted by the parameter $x_{c}$ and the rate at which the function transitions in controlled by a fourth parameter, σ. Determining the mathematical form of such a function is not straight-forward but is significantly easier if one considers the functional form of its derivative and integrate up to obtain the correct PDF. Figure 10 shows a cartoon illustration of a typical double power law and its derivative. In log-log space we can model the derivative of the function, $\frac{\mathrm{d}(\log{(f(x))})}{\mathrm{d}(\log{x})}$, as a hyperbolic tangent function constrained by the four parameters as follows:

19 $$ \frac{\mathrm{d}[\log{(f(x; \alpha , \beta , x_{c}, \sigma ))}]}{\mathrm{d}\phi} = \frac{\alpha - \beta}{2}\tanh{ \biggl( \frac{-\sigma (\phi - \phi _{c} )}{2} \biggr)} + \frac{\alpha + \beta}{2}, $$

where $\phi = \log{x}$ and $\phi _{c} = \log{x_{c}}$. Integrating Equation 19 with respect to ϕ and substituting x back in leads to the following function for the double power law PDF:

20 $$ f(x; \alpha , \beta , x_{c}, \sigma ) = K \frac{x_{c}^{\frac{\alpha + \beta}{2}}}{2^{\frac{\beta - \alpha}{\sigma}}} \biggl(\frac{x}{x_{c}} \biggr)^{\alpha} \biggl[1 + \biggl( \frac{x}{x_{c}} \biggr)^{\sigma} \biggr]^{ \frac{\beta - \alpha}{\sigma}}, $$

where K is a normalisation constant.

The normalisation of the function can be treated in two separate ways. The first method treats σ as a fixed value which tends to ∞, causing a sharp kink in the distribution at $x_{c}$ (Sharp Double Power Law, Sharp-DPL). The second method keeps σ as a variable parameter allowing for a smoother transition between power laws (Smooth Double Power Law, Smooth-DPL). The following sections detail how each separate method is treated.

#### A.5.1 Sharp Double Power Law

The Sharp Double Power Law (Sharp-DPL) is formulated by allowing $\sigma \rightarrow \infty $. The function in Equation 20 reduces to the following piece-wise function:

21 $$ f(x; \alpha , \beta , x_{c}) = K x_{c}^{\frac{\alpha +\beta}{2}} \left \{ \textstyle\begin{array}{l@{\quad}l} (\frac{x}{x_{c}} )^{\alpha}, & \quad x \leq x_{c} \\ (\frac{x}{x_{c}} )^{\beta}, & \quad x > x_{c}. \end{array}\displaystyle \right . $$

Equation 21 can be normalised analytically resulting in the following PDF:

22 $$ f(x; \alpha , \beta , x_{c}) = \frac{ (\alpha +1 ) (\beta + 1 )}{x_{c} [\beta -\alpha - (\beta +1 ) (\frac{x_{0}}{x_{c}} )^{\alpha +1} ]} \left \{ \textstyle\begin{array}{l@{\quad}l} (\frac{x}{x_{c}} )^{\alpha}, & \quad x \leq x_{c} \\ (\frac{x}{x_{c}} )^{\beta}, & \quad x > x_{c}, \end{array}\displaystyle \right . $$

provided $\alpha , \beta < -1$. It is not feasible to determine the maximum likelihood estimates of the three parameters (α, β and $x_{c}$) analytically so instead one has to perform a numerical optimisation of the log-likelihood function of the PDF. The CDF of the function is as follows:

23 $$ F(x; \alpha , \beta , x_{c}) = K' \left \{ \textstyle\begin{array}{l@{\quad}l} [ (\frac{x}{x_{c}} )^{\alpha +1} - ( \frac{x_{0}}{x_{c}} )^{\alpha +1} ] / (\alpha +1 ), & \quad x \leq x_{c} \\ \frac{ (\beta +1 ) [1 - (\frac{x_{0}}{x_{c}} )^{\alpha +1} ] + (\alpha +1 ) [ (\frac{x}{x_{c}} )^{\beta +1} - 1 ]}{ (\alpha +1 ) (\beta +1 )} , & \quad x > x_{c}, \end{array}\displaystyle \right . $$

where $K' = \frac{ (\alpha +1 ) (\beta + 1 )}{\beta -\alpha - (\beta +1 ) (\frac{x_{0}}{x_{c}} )^{\alpha +1}} $

#### A.5.2 Smooth Double Power Law

The Smooth Double Power Law treats σ as a variable parameter which introduces a smooth transition between separate power laws. Direct integration of Equation 20 introduces hypergeometric functions which makes the normalisation of the function difficult to approach. An alternative approach is to perform a numerical normalisation of the function by re-writing the PDF as follows:

24 $$ f(x; \alpha , \beta , x_{c}, \sigma ) = \frac{I (x; \alpha , \beta , x_{c}, \sigma )}{\int _{x_{0}}^{\infty }I (x; \alpha , \beta , x_{c}, \sigma )\mathrm{d}x}, $$

where

$$ I (x; \alpha , \beta , x_{c}, \sigma ) = \biggl( \frac{x}{x_{c}} \biggr)^{\alpha} \biggl[1+ \biggl( \frac{x}{x_{c}} \biggr)^{\sigma} \biggr]^{\frac{\beta -\alpha}{\sigma}}. $$

One can then determine the maximum likelihood estimates of all four parameters (α, β, $x_{c}$ and σ) using a combination of numerical optimisation of the log-likelihood of the above function and numerical integration of the stated integrand. The CDF is also obtained through numerical integration.
